# New drugs and polydrug use: implications for clinical psychology

**DOI:** 10.3389/fpsyg.2015.00267

**Published:** 2015-03-17

**Authors:** Antonio Iudici, Gianluca Castelnuovo, Elena Faccio

**Affiliations:** ^1^Department of Philosophy, Sociology, Education and Applied Psychology, University of PaduaPadua, Italy; ^2^Department of Psychology, Catholic University of MilanMilan, Italy; ^3^Psychology Research Laboratory, Istituto Auxologico Italiano IRCCS, Ospedale San GiuseppeVerbania, Italy

**Keywords:** polydrug use, drug combinations, drugs, clinical psychology, toxicology, new drugs

## Introduction: the phenomenon of polydrug use

The abuse of legal and illegal drugs is a complex and constantly evolving phenomenon. In recent years the polydrug use, term that refers to the use of two or more psychoactive drugs in combination to achieve a particular effect, has been on the rise. In many cases, one drug (amphetamine, cocaine, or heroin) is used as a base, with additional drugs being added to compensate for the side effects of the primary drug and to make the experience more enjoyable.

The traditional traffic of drugs, always cheaper and more often available on the Internet, is expanding to include medical prescription drugs that can be ordered without prescription, “smart” drugs (substances legally sold for other uses, such as fertilizers, bath salts, dietary supplements), and synthetic drugs (Cinosi et al., 2014). In Italy, in 2013, the Department of Drug Control Policy (DPA) has identified, made illegal, and seized more than 250 new substances, including synthetic cannabinoids, cathinones, phenethylamines, piperazines, and metossietamine (Dipartimento Politiche Antidroga Relazione annuale al Parlamento, 2013).

With the change in substances, the methods of recruitment and their use have also changed. The assumption is that combination of drugs increases the health risk much more compared to a single drug, e.g., alcohol and cocaine increase cardiovascular toxicity (Snenghi et al., 2015); alcohol or depressant drugs, when taken with opioids, lead to an increased risk of overdose; opioids or cocaine taken with ecstasy or amphetamines also result in additional acute toxicity. Benzodiazepines are notorious for causing death when mixed with other depressant substances, such as opioids, alcohol, or barbiturates (Kelly et al., 2015).

The unpredictability of the effect, due to the use of several substances randomly mixed, makes the substance use more attractive. Instead of looking for a specific effect, as was the case with the use of a single drug, polyusers seek the maximum alteration of consciousness and loosening of inhibitions (the so-called *garbage head syndrome*), which makes it much easier to achieve the euphoria.

The new phenomenology of the “culture” of the buzz calls for a re-thinking by the therapists and experts in the field in order to promote various forms of integration between professionals and to calibrate new methods of intervention.

## Problem statement

In the study of drug addiction, polydrug use is a complex phenomenon. Drug addiction research needs to be done by experts who have accurate knowledge of the effects of new substances and their interaction (which is difficult also for the toxicologist, given the variety of synthetic molecules recently introduced), the personal meanings linked to the use, and new contexts related to polydrug abuse. Biochemistry, pharmacology, but also psychology, sociology, and anthropology are the involved in the study of polydrug use.

What makes it difficult the network within experts (that is necessary in the equip group, and also in the collaboration between the public service, community service and psychotherapy) is the ability to refer to various professional skills and competences, recognizing the limits of their own knowledge and not superimposing one of the expertise over others, what happens for example considering the medical intervention, or considering the use or cessation of the substance use as a criterion of effectiveness of other not medical interventions.

This theoretical paper aims to present a new conceptualization of drug use, which complements that of “drug-addiction.” We will identify this different perspective using the term “toxico-philia.”

## Drug addiction and toxico-philia: alessandro salvini theoretical contribution

The label “drug addiction,” being composed by the words “drug” (e.g., *toxic*) and “addiction,” refers to biochemical and psychological features. This article proposes to distinguish the cases in which the behavior of an individual is the result of chemical compensation search to reward biochemistry, which is called “addiction,” from cases in which behaviors cannot be attributed solely to the biochemical mechanism, the so-called “toxicophilia” (Salvini, 2004). These terms conceptualize different aspects of substance use phenomenon, contiguous and not opposed.

“Drug addiction” refers to the strong need for a psychoactive substance necessary to maintain a physiological balance and reduce the abstinence symptoms. The intervention model based on the chemical balancing is probably the most effective in some particular cases, because it employs a set of procedures and pharmacological methods aimed at amending the organic equilibrium (Hawkins et al., 2002), e.g., drug methadone or antabuse. However, in many cases, the person does not reduce the consumption of other substances following the modification of biochemical mechanisms. Some people may stop using drugs suddenly, despite being declared in the “abstinence” phase and without using methadone. Alternatively, after detoxification, some people may immediately start using substances repeatedly; hence, the biochemical model is not sufficient to explain the consumption of substances nor their recurrence (Iudici, 2015).

This implies that other elements are necessary to understand the phenomenon, such as the perception of self and others with respect to the identity and the labeling of behavior. Common sense uses the adjective “addict” to identify consumers of substances, but this term refers to an individual with weak self-determination and poor cognitive maturity.

To understand how psychological elements affect drug use, the construct of “toxicophilia” was systematized. This term refers to the deliberate pursuit of the rewarding effects of one or more psychotropic substances, which are instrumental to generate changes in the psychological status, for example, change or confirm the idea of himself/herself, to live new sensations, share specific experiences within the group.

These mental states can help people achieve their desired goals, such as experiencing disinhibition with a partner, gambling, completing a tiring job or commitment, experiencing a mystical condition, dealing with painful experiences or, developing the curiosity and spirit of adventure derived from the effect of a mix of substances (which is not predictable), as in the case of polydrug use.

The construct of “tossicophilia” allows the researcher to represent drug users from a different perspective and reconstruct the meanings, intentions, and psychological journey that brought substance use.

## A new theoretical and methodological approach for clinical psychology: from the consumer labeling to the biographical career

In everyday language, drug users are called by the adjective “drugged” or “toxic,” and this term is often generalized to the whole person, promoting prejudice against the person in various roles and in various contexts. Consequently, this affects how a person perceives and represents him/herself.

Some scholars (the labeling theorists) have pointed out that deviance is not inherent in an act; instead, it reflects the tendency of majorities to negatively label minorities or those who deviate from standard cultural norms (Becker, 1963; Matza, 1969).

The deviant roles and the labels attached to them function as a form of social stigma (Faccio and Costa, 2013; Iudici et al., 2013). The attributions of some form of “pollution” or characteristics that mark the labeled person as different from others are always inherent in the deviant role. Society uses these stigmatic roles to them to control and limit deviant behavior: “If you proceed in this behavior, you will become a member of that group of people” (i.e., the “toxic group”).

Deviant roles powerfully affect how we perceive those who are assigned those roles. They also affect how the deviant actor perceives himself and his relationship with society (Faccio et al., 2013, 2014; Mannarini and Boffo, 2014, 2015).

Society uses the stigmatic label to justify its condemnation, and the deviant actor uses it to justify his actions. In this sense, “The deviant is one to whom that label has been successfully applied” (Becker, 1963, p. 9). “Instead of the deviant motives leading to the deviant behavior, it is the other way around, the deviant behavior in time produces the deviant motivation” (Becker, 1963, p. 29).

The repeated attribution to a role, such as deviant or addict, may increase the likelihood of relapse in drug abuse and enhance the self-perception of him/herself as deviant.

These processes lead indirectly to the creation of what Becker (1963) has called the “deviant career,” referring to the sequence of biographical events through which the individual becomes a greater expert in conforming to a certain role, that of the consumer of substances (Iudici and Maiocchi, 2014).

In contrast, the narrative approaches in clinical psychology and psychotherapy give great importance to the story, the narrative that the individual makes of his experience, in an effort to understand the different ways in which human beings inscribe the use of drugs in their biographies (Romaioli and Faccio, 2012).

## Conclusions

Substance abuse, especially polydrug use, is a very complex field of study. The simultaneous presence of medical, psychological, and social variables linked to the repeated substances use, manifold and different every time, has been identified by the expression: “*garbage head syndrome*.”

Although it is very important not to ignore this complexity, many professionals tend to simplify and prefer the medical reading of biochemistry at other levels involved, or the confusion between medical and psychological interventions.

In the case of polydrug use, it is even more important to investigate the linkage between the biochemical and the psychological components because the user often seeks a way to escape from reality rather than a specific feeling. The construct of tossicofilia can help clinical psychologists and social workers, as it allows them to understand the consumer world. Substance use can also be used as a strategy to deal with social events. The knowledge of these personal meanings is essential to the planning of a change and managing individual and grouping psychotherapy.

### Conflict of interest statement

The authors declare that the research was conducted in the absence of any commercial or financial relationships that could be construed as a potential conflict of interest.

## References

[B1] Becker H. S. (1963). Outsiders: Studies in the Sociology of Deviance. New York, NY: The Free Press.

[B2] Cinosi E. Corazza O. Santacroce R. Lupi M. Accivatti T. Martinotti G.. (2014). New drugs on the internet: the case of Camfetamine. BioMed Res. Int. 16:419026. 10.1155/2014/419026 25136586 PMC4124220

[B3] Dipartimento Politiche Antidroga Relazione annuale al Parlamento. (2013). Uso di Sostanze Stupefacenti e Tossicodipendenze in Italia. Roma: Governo - Presidenza del Consiglio dei Ministri Via della Ferratella in Laterano 51, 00184 - Roma.

[B4] Faccio E. Bordin E. Cipolletta S. (2013). Transsexual parenthood and new role assumptions. Cult. Health Sex. 15, 1055–1070. 10.1080/13691058.2013.806676 23822798

[B5] Faccio E. Casini C. Cipolletta S. (2014). Forbidden games: the construction of sexuality and sexual pleasure by BDSM ‘players.’ Cult. Health Sex. 16, 752–764. 10.1080/13691058.2014.909531 24828811

[B6] Faccio E. Costa N. (2013). The presentation of self in everyday prison life: reading interactions in prison from a dramaturgic point of view. Glob. Crime 14, 386–403. 10.1080/17440572.2013.83176124828811

[B7] Hawkins J. D. Catalano R. F. Arthur M. (2002). Promoting science-based prevention in communities. Addict. Behav. 90, 1–26. 10.1016/S0306-4603(02)00298-8 12369478

[B8] Iudici A. (2015). Health Promotion in Schools: Theory, Practice and Clinical Implications. New York, NY: Nova Pubblisher.

[B9] Iudici A. Maiocchi A. (2014). Community justice and juvenile offender: the management of an individual case with criminal slope with community involvement. Mediterr. J. Soc. Sci. 5, 2015–2027 10.5901/mjss.2014.v5n20p2015

[B10] Iudici A. Vallorani M. Antonello A. (2013). Innovative law old services: application and limitations in the application of restorative justice in italy: description and analysis of a case study. Int. J. Innov. Appl. Stud. 4, 43–51.

[B11] Kelly A. B. Evans-Whipp T. J. Smith R. Chan G. C. K. Toumbourou J. W. Patton G. C.. (2015). A longitudinal study of the association of adolescent polydrug use, alcohol use and high school non-completion. Addiction. [Epub ahead of print]. 10.1111/add.12829 25510264 PMC4361375

[B13] Mannarini S. Boffo M. (2014). An implicit measure of associations with mental illness versus physical illness: response latency decomposition and stimuli differential functioning in relation to IAT order of associative conditions and accuracy. PLoS ONE 9:e101911. 10.1371/journal.pone.0101911 25000406 PMC4084979

[B12] Mannarini S. Boffo M. (2015). Anxiety, bulimia, drug and alcohol addiction, depression, and schizophrenia: what do you think about their aetiology, dangerousness, social distance, and treatment? A latent class analysis approach. Soc. Psychiatry Psychiatr. Epidemiol. 50, 27–37. 10.1007/s00127-014-0925-x 24972643

[B14] Matza D. (1969). Becoming Deviant. Englewood Cliffs, NJ: Prentice Hall.

[B15] Romaioli D. Faccio E. (2012). When therapists do not know what to do: informal types of eclecticism in psychotherapy. Res. Psychotherapy Psychopathol. Process Outcome 15, 10–21.

[B16] Salvini A. (2004). La tossicofilia in Droghe, eds Salvini A. Zamperini A. Testoni I. (Torino: Utet), 336.

[B17] Snenghi R. Forza G. Favretto D. Sartore D. Rodinis S. Terranova C.. (2015). Underlying substance abuse problems in drunk drivers. Traffic Inj. Prev. 16, 435–439. 10.1080/15389588.2014.968656 25436517

